# Determinants of received care time among Finnish home care clients and assisted living facility residents: a time-motion study

**DOI:** 10.1186/s12877-024-05355-w

**Published:** 2024-09-12

**Authors:** Tiina Pesonen, Visa Väisänen, Mari Aaltonen, Johanna Edgren, Laura Corneliusson, Salla Ruotsalainen, Timo Sinervo

**Affiliations:** 1 Finnish Institute for Health and Welfare, Helsinki, Finland; 2 Department of Health and Social Management, University of Eastern Finland, Kuopio, Finland

**Keywords:** Care time, Home care, Assisted living, Older people, Functioning

## Abstract

**Background:**

Ageing populations and care workforce shortages across Europe are causing challenges for care services for older people. Therefore, it is paramount that limited care resources are allocated optimally, based on the clients’ care needs. Multiple functioning-related factors have been identified that determine the amount of care time clients receive, while organizational and other factors remain largely unexplored. The aim was to examine how various individual and organizational factors are associated with clients’ received care time in different care settings.

**Methods:**

Cross-sectional observational study design with data from time and motion study, registers, and surveys was used. In total, 1477 home care clients and 1538 residents from assisted living facilities with 24/7 service participated, from 61 Finnish care units. Linear mixed-effect modeling was used to examine the association between individual and organizational-level variables and received care time.

**Results:**

Physical functioning was the strongest predictor of received care time in both care settings. In home care, greater pain, more unstable health, and higher team autonomy were associated with increased care time. In assisted living, depressive mood and higher staffing level of the organization were associated with care time. Clients who received informal care also received significantly more care time from nurses in both care settings.

**Conclusions:**

Physical functioning was the main driver of received care time. Interventions that maintain or improve physical functioning can help restrain the growing need of care resources, although it is important to ensure that each client receives care according to their holistic care needs.

## Introduction

In Finland, care services for older people have undergone several changes over the past decades, as the ageing policy has shifted to emphasizing home care as the priority option for older people needing regular care [1]. Simultaneously, institutional care has decreased and is replaced by assisted living facilities with 24-hour assistance [2]. A similar trend is seen in other Nordic countries, where long-term care and nursing services are predominantly shifting from institutions to home-based care in municipalities [3]. The population is aging rapidly, which means that the number of older people, especially the very old is increasing. Since the supply of round-the-clock care has not grown at the same pace, the number of older people receiving particularly home care, is increasing [4], although the coverage of home care has decreased in recent years, even among those over 85 years old [5]. In 2021, 16% of the Finnish population over 75 received home care and 7% received round-the-clock care [6]. Meanwhile, care services for older people suffer from a workforce shortage [7, 8] and a substantial share of staff in home care and assisted living report that the workload is too high [5, 9]. Similar challenges exist throughout Europe, as aging populations and staff shortages in health- and social care services cause major concerns [10].

In Nordic countries municipalities are responsible for providing services for older people [3]. The situation was the same in Finland during the data collection of this study, although after the national health and social reform in 2023, the responsibility for organizing public health, social, and rescue services was transferred from municipalities to new wellbeing services counties [11]. The municipalities are highly autonomous, which may lead to significant differences between them. For example, eligibility criteria, service coverage, and spending on older care services may vary between municipalities in all Nordic countries [12]. In Finland, as in other Nordic countries, older care services are publicly funded by municipal and state taxes in addition to user fees [11]. However, public spending on older care services varies between Nordic countries and is the lowest in Finland in relation to GDP (Gross domestic product) [11].

In Finland, home care combines home nursing and home help services, and most units operate in the public sector [13]. However, there are some private providers, both for-profit companies and non-profit organizations. The user charges for home care are regulated (734/1992) and they are determined by the number of service hours, the client’s monthly income, and the size of the household [13]. The municipality can also give out service vouchers that can be used to purchase services from predetermined service providers [13]. The private sector (mostly for-profit companies), on the other hand, provides more than half of assisted living services (24/7 service) in Finland [14]. Similarly, the share of the private sector is high in Sweden [12, 15], unlike in Denmark and Norway. Also, in assisted living services the user fees are determined by law (734/1992) and they are based on the resident’s monthly income, and like home care, municipalities can admit a service voucher for the client. Majority of care workers in Finland (approx. 70–75%) in both care settings are practical nurses, which are mainly responsible for assisting older clients in activities of daily living [16]. Practical nurses in Finland are licensed health and social care professionals with three years of curriculum-based vocational education [17].

The quality of care and staff shortage in services for older people have caused national concern. Higher amount of care time clients receive has been shown to be relevant for quality of care [18] as well as sufficient staff resources [19–22]. To improve the quality of care and to increase clients’ received care time, the Finnish government has regulated a minimum nurse-patient ratio for long-term care facilities [23]. The nurse-client ratio became mandatory in October 2020, with the required number of nurses per patient being 0.5. The ratio has gradually increased to 0.65, with the current government planning to reduce it to 0.60 from the beginning of 2025 [23, 24].

The amount of care time home care recipients and assisted living residents, both hereafter referred to as clients (for clarity and in line with enabling active care participation [25]), receive is associated to numerous factors on the individual, policy-, organizational-, and operational levels. Previous care time studies have focused on individual-level factors related to clients’ received care time in care services for older people. The results of these studies show that particularly decreased physical functioning is associated with the increased amount of care time received [26–29]. However, individual-level factors, such as the client’s clinical and functional status, only seem to explain part of the total received care time. Differences between municipalities may explain variation in care services for older people, in addition to differences within the municipalities [30]. Several studies have shown that care times vary notably between work units [27, 31, 32], which may indicate differences in staffing, management, or work practices. The main organizational factor explaining the differences in care time between work units is the staffing level.

According to McGregor et al. (2005) higher staffing level was associated with not-for-profit facility ownership in Canada. Similar results were found in a US study, where for-profit chains had fewer registered nurses and lower total nurse staffing hours compared to government facilities [33]. The results of a Swedish study also showed that public nursing homes had higher staffing level and a higher proportion of advanced competence, defined as employees with a college or university health care education, than private nursing homes [15]. However, in addition to staffing levels, it is also important to consider how the work is organized. One common approach to organize care more effectively is to use enterprise planning systems (ERP) that divide care work into small operations, which can lead to high operational efficiency [34]. But it may also lead to an assembly-line type of work, which can in turn lead to higher stress and lower job satisfaction. Another almost contradictory development is the use of self-organizing teams, which have been identified to increase job satisfaction and job retention [35, 36], as well as client satisfaction and, possibly, productivity [37]. Increasing the proportion of customer-specific work in employees’ working days is one way to improve productivity, leading to higher care time with fewer employee resources. It thereby seems that other organizational factors are important in explaining variation in clients’ received care times.

In the current situation, where the number of clients is growing and the workforce shortage is worsening, the need for optimal allocation of limited care resources is paramount. Older people have varying care needs, which require a person-centred approach. The World Health Organization defines person-centred care as ensuring that services are tailored to people’s needs and are provided in partnership with them [38]. Person-centred care has positive impacts on care staff, clients, their relatives and other informal care givers [39–41] and it has become a key care strategy in services of older people [42]. Self-organizing teamwork is based on the principles of person-centred care as it emphasizes tailoring the care for clients’ needs and care continuity, as the employees are familiar with the clients and vice versa [43]. Person-centred provision of care and its alignment with client’s care needs is stipulated in Finnish law (980/2012, 565/2020, 14§). There however seems to be little information on how well these aims are being met.

High-quality and holistic care requires identifying the factors associated with the clients’ received care time. Exploring factors associated with care time in different care settings may help evaluate if resources are being allocated justly. Furthermore, as the amount of care time is relevant to the quality of care, this study may reveal previously unknown factors that influence received care time. The results can be utilized in developing care services, allocating care resources, and improving work practices, which can all lead to improvements in the quality of care or efficiency of services for older people. For example, providers can better analyse the client structure of the units, especially accounting for the individual factors related to the client’s care time, when allocating care resources. The results can also inform policymakers and care service managers to consider different ways to manage the growing need for services and resources in older care services. Therefore, aims of this study were to explore individual-level as well as organisation-level factors associated with received care time within care services for older people. The research questions were:

1. Which individual and organisational level factors are associated with the clients´ received care time in home care services?
2. Which individual and organisational level factors are associated with the clients´ received care time in assisted living facilities with 24-hour assistance?

## Methods

### Design

Cross-sectional observational study design with data from time and motion method, registers and survey were used. Time and motion method is useful for identifying activities and measuring the time for each activity when providing care to the client [44]. The care workers’ work was divided into tasks, and the time spent on the activities during the day was self-reported using paper forms. Time and motion data were merged with data from registers and survey.

### Participants and data collection

Home care units and assisted living facilities with 24-hour assistance (hereafter assisted living) using the Resident Assessment Instrument (RAI) were invited to the study. Data were collected in total from 17 publicly funded and publicly provided home care units and 44 assisted living facilities, of which 17 (39%) were private (for-profit). The units were located both in urban and rural areas, in 15 different wellbeing services counties (out of 22).

Time-motion study was conducted between 11.-24.10.2021 with a study period of one week in one unit. Internal care workers (employees working in a certain work unit) in homecare and assisted living facilities documented their work time per task for 7 days in home care and for 24 h in assisted living. Therapists and external workers (employees working for more than one work unit or working as employees of another organization) documented their care time for a week in assisted living. Previous similar time and motion studies have used a 24-hour/7-day observation period, which has been found to be adequate for reliability [27]. The forms contained the client’s name, care task’s start and end times, and care task’s category.

In this study, care time refers to both direct and indirect care time. Direct care time is time that was spent with the client. Indirect care time is client-specific work where the client was not present, such as nursing documentation and arranging services for the client [45]. Indirect care supports direct care and is crucial in comprehensive care. Indirect care was included in the total care time to account for the whole range of care-related worktime that is aimed at specific client, regardless of whether the client is present or not.

To obtain the total care time received per client, the direct and indirect care worker-level data were grouped by the client ID. The data were merged with the clients’ latest RAI assessments from the Finnish RAI-register for clients’ functional status and background information. The RAI is used to evaluate clinical and functional status and need for care of older people, as well as quality of care [46]. The RAI provides validated individual-level indicators on, for example, performance in activities of daily living, depressive mood, health stability, cognition, and need for care and services. The RAI system, which is used in several countries, has been voluntarily used in Finnish care services for older people for more than 20 years [47]. In 2023, it became mandatory (980/2012, 15 a §) for assessing the care needs of older people in regular home care service or other long-term care service in Finland. Assessments are performed for the client twice a year or more often if there are changes in the client’s condition.

Family members or acquaintances voluntarily also reported the time spent with the client during the week, which was interpreted as informal care in this study. However, informal care was not included in care time as the study focused on formal care time received from care professionals. Similarly, home support services (such as meal or grocery services) and attendance in day activities for older people were not included in care time.

Information on team independence was collected by an online survey for the managers of the care units. To measure the ratio of care staff to residents, staffing level data were extracted from an open national database [48]. Data are collected twice a year (spring and fall) and in this study data from March 2022 were used.

### Study variables

*The dependent variable* was wage-adjusted care time received per day. Care time included direct and indirect care received from all care workers in the organisation, in addition to care time received from external workforce, such as physicians and physical therapists. External home visits by physicians and therapists for home care clients were retrieved from the Finnish Care Register for Health Care. Care time of group activities and other actions with multiple clients was divided equally between all participating clients.

To account for limited care resources, care time was wage adjusted by occupation based on Finnish wage statistics. The care time of practical nurses was weighed as 1.0, registered nurses as 1.15, and physicians as 2.50. Since the majority of care time was from practical nurses, the wage adjusting did not have a significant effect on care time.

#### Independents variables

##### Individual-level

For individual-level variables, multiple RAI indicators were used. For all RAI indicators, a higher value indicates lower performance or functioning.

Physical functioning was measured using ADL-H (Activities of Daily Living Hierarchy), which measures performance in activities related to eating, personal hygiene, toilet use, and locomotion (scale 0–6) [49] and IADL (Instrumental Activities of Daily Living Capacity scale), which measures performance in instrumental activities of daily living via three items: meal preparation, phone use, housework (scale 0–6). IADL was only available for home care clients [50].

CHESS (Changes in Health, End-stage disease, and Symptoms and Signs) measures the stability of health: clients with higher values are more likely to be admitted to a hospital or emergency department (scale 0–5) [51].

Depressive mood was measured using DRS (Depression Rating Scale). Scale 0–14, with values of 3 or more referring to a high likelihood of depression. The indicator was used as a binary variable (0–2 and 3 or more) [52].

CPS (Cognitive Performance Scale) measures the level of cognitive function, with a value 6 meaning comatose or not present (scale 0–6) [53].

PAIN measures the frequency and intensity of pain the resident experiences (scale 0–3) [54].

Problem behaviour refers to antisocial behaviour or aggressiveness. Binary variable was determined from MAPLe (Method for Assigning Priority Levels) indicator with groups 46 and 54 (behavioural problems) defined as disruptive behaviour [55].

Additionally, whether the client had received informal care during the study period was measured as a binary variable.

##### Organisation-level

Team independence was measured as a sum variable, which consisted of seven questions (scale 1–5), on whether the teams/workers could affect planning of shifts, division of care work, recruitment, the need and recruitment of substitute workers, ways of working, care planning, and professional development. Value of Cronbach’s alpha was 0.76 in home care and 0.58 in assisted living.

Staffing level and whether the organisation was public or private were used as organisation-level variables in assisted living. Staffing level indicates the number of care staff in relation to the number of clients (nurse-patient ratio). The variables were only used in assisted living, as staffing level information was not available for home care and all home care organisations in the study were public.

For background variables, age, sex, and the municipality of the organisation were used.

### Data analysis

Descriptive statistical analysis was performed to explore the characteristics of the clients in home care and in assisted living facilities.

To analyse the associations between wage-adjusted care time and the independent individual-level and organisation-level variables, univariate regression analysis and linear mixed-effect modelling were utilized. Due to the inclusion of organizational-level variables, the assumption of independent observations in linear regression is violated. Therefore, linear mixed-effect models were used with municipality as a random effect and all other variables as fixed effects. While the number of municipalities in both care settings was low (Home care *n* = 10; Assisted living *n* = 23), Maas & Hox (2005) have shown that mixed-effect models with small number of groups have reliable regression coefficients and standard errors [56]. In addition, several other studies have indicated that a small number of clusters does not necessarily impact the accuracy of predictors [57, 58]. As the study focuses on the effects of explanatory factors on the amount of received care time, rather than aiming to explain the municipality-level variance in care times, the use of mixed-effect modelling was seen as appropriate.

The mixed-effect models were adjusted for age and sex. P-values were calculated using the Satterthwaite’s degrees of freedom method [59] and the method outlined in Nakagawa & Schielzeth (2012) was used to estimate percentage of variance in received care times explained by the mixed-effect models (R^2^). Marginal R^2^ measures the effects of fixed effects, while conditional R^2^ takes into account both the fixed and random effects [60].

Statistical significance was defined at *p* < 0.05. All statistical analyses were performed using R Statistical Software version 4.2.2 for Windows 10 [61]. Mixed-effect modeling was conducted using packages lme4 [62] and lmerTest [63].

### Ethical considerations

Finnish Institute for Health and Welfare Ethical Review Board approved the study (THL/1447/6.02.01/2021). Participation in the study was voluntary for the units. Care workers documented their work time as part of their regular work and reporting the informal care time was voluntary for family members. All participants were informed in detail about the study and their rights in an information sheet. Filling and returning the surveys was seen as informed consent to participation. The study was conducted according to ethnical principles of the Declaration of Helsinki [64].

## Results

Characteristics of the study participants are presented in Table 1. The majority (67–68%) of the participants were female and they had been in the care services for on average three years. Received care time was on average 38 min (median: 28; variance: 1-380) in home care and 117 min (median: 110; variance: 7-503) in assisted living facilities. In both care settings, the majority of the care time was direct care time (33 min in home care and 90 min in assisted living).

Home care clients had on average higher functional status than residents in assisted living facilities. Performance in activities of daily living and cognitive functions were significantly lower among residents in assisted living compared to home care clients. Behavioural symptoms and depressive mood were significantly more common in assisted living facilities. The average team autonomy was at the same level in both care settings.

**Table 1:** Characteristics of study participants by type of care setting

	Home care *n* = 1477		Assisted living facility *n* = 1538	
Variable	Mean (SD)	95% CI	Mean (SD)	95% CI
**Received care time (minutes)**	38.1 (35.3)	36.3–39.9	116.9 (51.7)	114.3–119.4
**Age**	81.4 (10.2)	80.9–81.9	83.3 (9.0)	82.9–83.8
**Sex (female)**	66.6%	64.1–67.0	67.7%	65.3–70.0
**Years as a client**	3.2 (3.3)	3.1–3.4	3.1 (3.2)	2.9–3.2
**Received informal care (yes / no)**	23.6%	21.5–25.8	25.5%	23.3–27.7
**RAI indicators**:				
**ADL-H (0–6)**	0.8 (1.2)	0.7–0.8	3.8 (1.7)	3.7–3.9
**IADL (0–6)**	2.7 (1.9)	2.6–2.8	5.7* (0.6)	5.6–5.8*
**CHESS (0–6)**	1.0 (1.1)	1.0–1.1	1.4 (1.2)	1.3–1.5
**CPS (0–6)**	1.5 (1.2)	1.4–1.5	3.3 (1.6)	3.2–3.4
**PAIN (0–3)**	0.7 (1.0)	0.6–0.7	0.7 (0.9)	0.7–0.7
**Depressive mood (%)**	11.9%	10.3–13.6	28.0%	25.7–30.2
**Behavioural symptoms (%)**	14.6%	12.8–16.4	44.6%	42.1–47.1
**Team autonomy (1–5)**	2.7 (0.5)	2.7–2.8	2.8 (0.3)	2.8–2.9
**Private organisation (%)**	0%		38.4%	35.9–40.9
Staffing level**			0.65 (0.1)	0.65–0.66

* *n* = 167: Assisted living facilities in some municipalities use the RAI-HC tool, which includes IADL

** Data not available for home care

Indicators: ADL-H = Activities of Daily Living, IADL = Instrumental Activities of Daily Living, CHESS = Changes in Health, End-stage disease, and Symptoms and Signs, CPS = Cognitive Performance Scale, PAIN = Pain scale. Lower value indicates better functioning

**Fig. 1:**
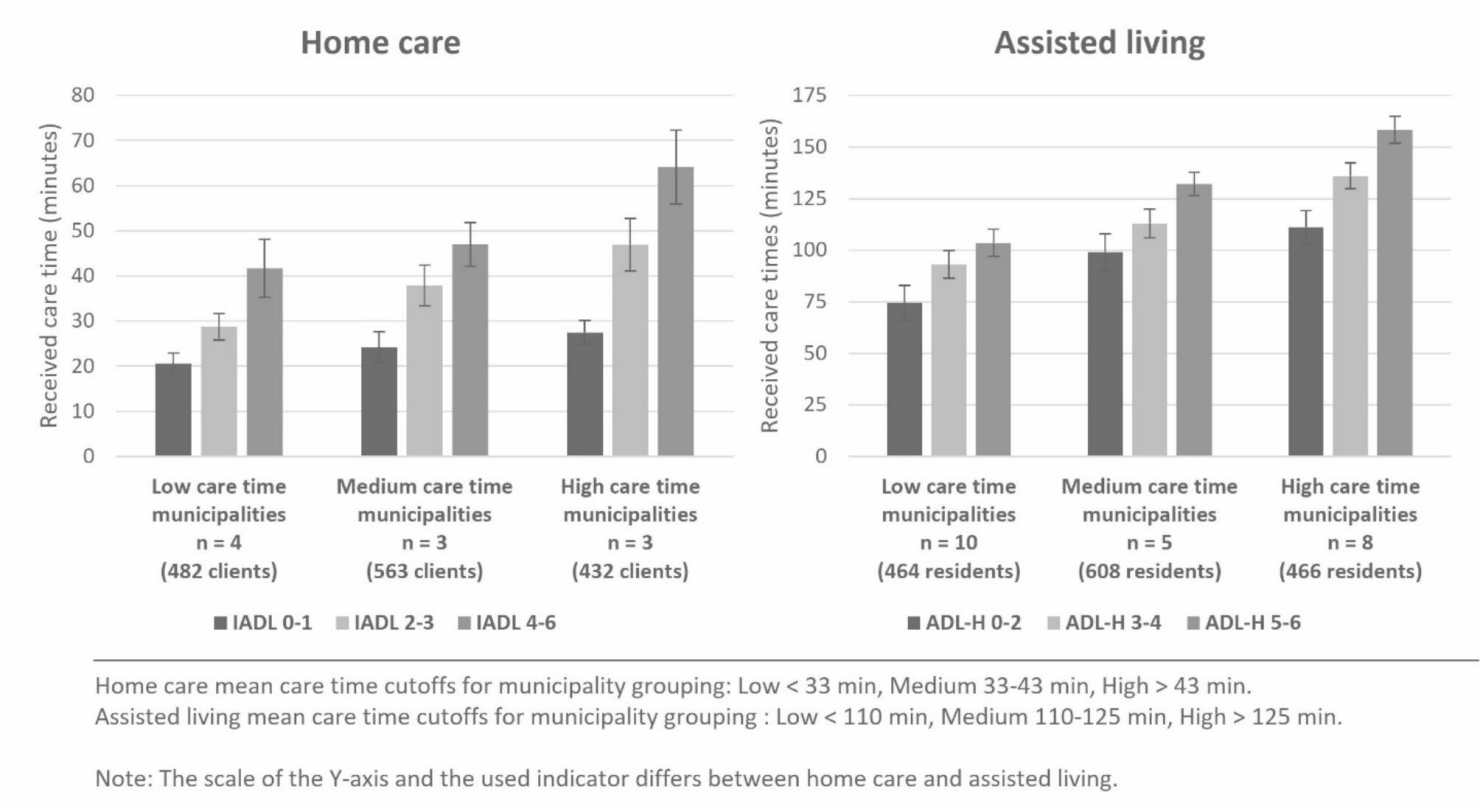
Average received care time by municipality and IADL in home care and ADL-H in assisted living facilities. Municipalities were grouped to low, medium, and high care time by the clients’ average received care time received in relation to the average care time of the care setting

The received care time varied significantly between municipalities (Fig. 1). Care time differences between municipalities were proportionally higher in home care compared to assisted living facilities. Additionally, in both care settings, the municipality-level variance of received care time was higher among clients with lower function in IADL/ADL-H.

The null mixed-effect models indicated that municipalities explained 4% of variation in care time in home care and 23% in assisted living facilities.

In home care, all RAI indicators and receiving informal care were statistically significantly associated with increased received care time in univariate models, with increased ADL-H and PAIN scores and depressive mood being the strongest predictors (Table 2). In the adjusted multivariate mixed-effect model, ADL-H score remained the strongest predictor of received care time. Increases in IADL, CHESS, and PAIN scores and receiving informal care were moderately associated with increased care time. Importantly, clients whose carers worked in teams with higher team autonomy, appeared to receive on average more care time while controlling for the functioning of the client, although the effect was slightly non-significant.

In assisted living, higher ADL-H, CPS, and PAIN scores, and receiving informal care were associated with increased care time in univariate models. For organisational variables, higher staffing level and public ownership were associated with higher care times. In the adjusted multivariate mixed-effect model, ADL-H remained the main predictor of received care time alongside depressive mood. In addition, receiving informal care and being a client of an organization with a higher staffing level of the organisation were statistically significantly associated with care time, while accounting for the RAI indicators. Furthermore, residents in private organisations appeared to receive less care time, even when controlling for other variables such as the functioning of clients or staffing level.

For background variables, older age was associated with receiving more care time in home care, but less time in assisted living. In home care, female sex was associated with higher care time. Notably, regional variance in care provision was a major driver of the care time received by clients, especially in assisted living. In assisted living, fixed effects (dependent variables) explained only 13% of the variance in care time, while both fixed and random effects (municipality) together explained 27% of the variance. In home care, fixed effects explained 21% of the variance in care time, while both fixed and random effects explained 31% of the variance.

**Table 2:** Univariate linear regression and multivariate mixed-effects analysis on the associations of individual and organization-level factors on clients’ received care time in home care and in assisted living

	Home care		Assisted living facility with 24-hour assistance	
	Univariate models	Multivariate mixed-effect model	Univariate analysis	Multivariate mixed-effect model
Variable	Estimate (95% CI)	Estimate (95% CI)	Estimate (95% CI)	Estimate (95% CI)
**ADL-H (0–6)**	11.24 (9.85–12.62) ***	8.43 (6.80-10.06) ***	9.60 (8.16–11.03) ***	8.95 (7.21–10.69) ***
**IADL (0–6)**	6.10 (5.20–7.01) ***	2.30 (1.23–3.38) ***		
**CHESS (0–5)**	6.07 (4.41–7.73) ***	1.83 (0.18–3.49) *	0.58 (-1.53-2.70)	-0.88 (-3.01-1.25)
**CPS (0–6)**	5.41 (3.96–6.87) ***	0.15 (-1.43-1.73)	4.58 (2.97–6.19) ***	-0.04 (-1.91-1.83)
**PAIN (0–3)**	7.02 (5.22–8.82) ***	2.84 (1.05–4.62) **	5.52 (-1.71-4.31) ***	^
**Depressive mood**	13.18 (7.66–18.69) ***	4.50 (-0.47-9.74)	4.75 (-1.02-10.51)	7.81 (1.85–13.77) **
**Behavioural symptoms**	7.53 (2.45–12.61) **	0.29 (-4.62-5.20)	-0.98 (-6.18-4.23)	-4.51 (-9.85-0.83)
**Team independence (1–5)**	3.78 (-0.36-7.92)	5.03 (-0.06-10.12)	1.71 (-6.50-9.91)	0.43 (-10.61-11.47)
**Private organisation**			-28.1 (-33.28 - -22.97) ***	-13.05 (-22.13- -3.97) ***
**Staffing level**			0.56 (0.18–0.94) **	0.48 (0.04–0.91) *
**Received informal care (yes)**	13.43 (9.25–17.61) ***	10.54 (6.56–14.53) ***	10.85 (4.93–16.76) ***	6.77 (0.76–12.78) *
**Age**	0.35 (0.18–0.53) ***	0.19 (0.03–0.36) *	-0.55 (-0.84- -0.26) ***	-0.32 (-0.61- -0.03) *
**Sex (female)**	6.1 (2.30–9.90) **	5.02 (1.50–8.55) **	-5.29 (-10.82-0.24)	-4.13 (-9.52-1.25)
		Marginal R^2^ = 0.21 / Conditional R^2^ = 0.31		Marginal R^2^ = 0.13 / Conditional R^2^ = 0.27

* = *p* < 0.05; ** = *p* < 0.01; *** = *p* < 0.001

^ = PAIN was omitted from the assisted living facility multivariate model (when included, *p* = 0.952), as it significantly reduced the performance of the model due to multiple missing values (*n* = 54)

Intraclass correlations (ICC): Home care null model: 0.04, multivariate model: 0.12. Assisted living null model = 0.23, multivariate model: 0.16

Note1: Models adjusted for age and sex. Dependent variables as fixed effects and municipality as random effect

Note2: IADL not available in assisted living. Staffing level and private organization not available in home care

Indicators: ADL-H = Activities of Daily Living, IADL = Instrumental Activities of Daily Living, CHESS = Changes in Health, End-stage disease, and Symptoms and Signs, CPS = Cognitive Performance Scale, PAIN = Pain scale. Lower value indicates better functioning

## Discussion

Functional status, as measured by activities of daily living and instrumental activities of daily living, was the main predictor of received care time in both care settings. This result is in line with previous studies, which indicate that most care time in care services for older people is used for helping with activities of daily living [26–29]. In addition, we had similar findings compared to Norwegian study, where the older adult’s functional decline was associated with allocation of more hours of help, even when other variables were controlled for [30]. However, decline in functional ability, measured with ADL-H and IADL, is associated with decline in other areas of functioning [65, 66]. For example, cognitive decline has a substantial impact on functional status, as well as depression, which reduce physical activity [67, 68]. In addition, depression [69] and behavioural symptoms [70] are more common in people with cognitive difficulties. Depression is also associated with pain, aggression, and quality of life [71, 72]. Our results may reflect this accumulation of health problems and reduced functioning as increased care needs related to activities of daily living.

Based on our study, clients with more severe pain received more care time in both care settings. Significant pain often reduces clients’ quality of life and physical activity and increases depression and aggression [71, 73, 74]. Therefore, clients with more pain may need more care resources. Frailty of older people often leads to hospitalization, mortality, and increased care needs [75], which is in line with our findings, as home care clients with unstable health received more care time.

Care time was mainly driven by performance in activities of daily living and from the point of quality of care, it is essential that persons with lower functional ability receive the required amount of care time. However, from the perspective of managing the growing need of services and care resources in older care services, slowing the rate of decline in these functions can help restrain care needs and the costs of services for older people. Therefore, care services should aim to prevent functional decline whenever possible, as several individual factors affect physical activity and functioning. One way to maintain physical activity and reduce care needs is to increase rehabilitation and rehabilitative care in home care and assisted living facilities, even though rehabilitative work can take more time from the carers [76]. Another way is to focus on factors that may prevent cognitive decline and thus help maintain physical function, such as physical exercise, a healthy diet, and social activity [77–79]. In addition, it would be important that those who already have a dementia diagnosis receive rehabilitation that maintains their remaining functioning. Also, effective pain management should be part of the daily care for clients who need it, to maintain physical activity and improve the overall quality of care. Finally, social activity should be supported, and the client’s remaining resources should be utilized when planning personally meaningful activities that support person-centred care.

However, health promotion and risk prevention for older persons is reasonable to some extent, but not infinitely. Improving or maintaining physical functioning is not always possible, and during the last year of life majority of older people will need substantial support and care. It is essential to ensure that also then the allocated care time meets the client’s care needs. Organizations should further utilize the current client structure in planning care provision and resourcing. Higher resources must be allocated to the units with more demanding client structures to ensure holistic and high-quality care, regardless of the prevailing staffing level regulation.

Based on our findings, the autonomy of care teams should be encouraged, especially in home care, as it appears to positively influence received care time of clients. Self-organizing care teams might have better care planning and more innovative ways of working, which can increase care time given to clients. Care provided by autonomous teams can also be more person-centered [80], and employees might be able to tailor their work according to clients’ needs instead of performing routine tasks. Previously self-organizing teams have been associated with higher job satisfaction and workforce retention, in addition to better patient outcomes [36, 81]. Higher team autonomy can also indirectly affect care time through reputation and the consequent success in recruitment. Therefore, further applications of self-organizing teams should be explored.

One notable result was that clients who had received informal care during the study period had on average significantly more care time in both care settings, even when accounting for care needs. One possible explanation can be active family members or acquaintances, who demand better or more frequent services. Additionally, all RAI assessments might not have been up to date, or the chosen indicators did not measure all facets of care needs, which informal care can complement. It is possible that the increased presence of relatives signifies worsening functional status or end-of-life care, which leads to higher care needs.

Received care time had more variance in home care compared to assisted living facilities. Residents in assisted living were more homogeneous in their characteristics and functional status, and the care in home care is organized through client visits, which become more frequent as care needs increase. In assisted living the connection between care needs and care time seems to be more indistinct. Care in assisted living also includes more group activities, which had their time shared equally between participating residents. In addition, the results can highlight care policy, where the need for care is mainly determined based on the performance of daily activities, while cognitive and social aspects might only receive minor attention. Lastly, the results may reflect the current environment where care resources and care personnel have accumulated in assisted living, due to the recent staffing level not applying to home care units [82].

Municipality of the organization strongly affected clients’ received care times. It is possible that due to high autonomous of municipalities [3] and the lack of national criteria, care eligibility varies regionally and between municipalities, which raises questions on equity of care. Similar signs of care inequality in home care were found in a previous study conducted in six European countries [83]. Other factors, such as the current staffing level in assisted living, varying resources allocated to home care, the availability of care workforce, and different ways of working (e.g., rehabilitative work), can also affect variance in care times between municipalities. Despite regulation, staffing level seemed to have an effect on care time, which remained when controlling for client’s functioning and other factors. In addition, clients in privately owned assisted living facilities received less care time, even in the fully adjusted models, a dynamic that has been found in previous studies [33, 84]. While it is possible that lower care times can stem from better average functioning of clients, the difference in care times remained in the adjusted model, which indicates that client characteristics do not explain the discrepancy in care times between public and private assisted living facilities. As such, more oversight might be needed to ensure that all clients, regardless of their care unit, receive sufficient care.

Residents in assisted living might receive more care time related to social activity than clients in home care. Abdi et al. (2019) identified three main areas where older people need care and support: (1) social activities and relationships; (2) psychological health; and (3) activities related to mobility, self-care, and domestic life [85]. As our findings indicate that the amount of care time stems mainly from activities related to mobility, self-care, and domestic life, the question remains whether current care meets the social and psychological needs of the clients, especially in home care. It is possible that the current care of older people primarily aims to meet the clinical needs of the clients. According to a previous Finnish study, the care time in relation to the client’s clinical needs was sufficient, but not necessarily perceived as appropriate, due to the lack of psychosocial aspect [9]. Therefore, the amount of care time that especially supports social functioning, should be researched in the future. Lastly, it is important to note that functioning varies between clients, and while measures to lower the amount of care needed can mitigate current challenges in care, the time clients receive should always meet their care needs.

### Limitations

This study had some limitations. First, this study used self-reporting surveys for the care workers, which can lead to overreporting of care time and to discrepancies in how workers interpret and fill the surveys [86]. In time motion studies, data produced by the subjects, as opposed to observers, can reduce reliability [86]. However, the workers reported the start and end times of each task, which may decrease the risk of overreporting. Additionally, the surveys used were relatively complex, and while video tutorials and training sessions were arranged for the participating units, it is likely that for example the amount of temporary workforce affected the data collection, which can reduce the amount of care time. Furthermore, the sum variable of team independence suffered from poor internal consistency in assisted living (0.58), which can possibly affect the results, for example by lowering the model fit or biasing the estimates. However, it is also possible that the independence of care teams does not significantly vary in this care setting (compared to home care), which could result in poor functioning of the scale.

Second, as participation in the study was voluntary, some selection bias is likely. Multiple care units that initially signed up for the study ended up not participating due to challenges with COVID-19 or the availability of the care workforce. Therefore, participating units might have had on average better COVID-19 or care workforce situation, which can influence the overall care time. During the data collection, there were regional COVID-19 restrictions in place, which might have affected some care units differently.

Third, all RAI assessments used might not have been up to date. While the policy is to assess clients every six months and when changes in functional status occur, some clients might have had outdated assessments, which can reduce the statistical associations to care time. Additionally, as the staffing level information was not available for the data collection period, the nearest complete data was used (February 2022). This can affect the results if some care units have undergone significant changes in their nurse-to-patient ratio. Fourth, care time in this study did not include information on attendance in day activities and home care support services. Additionally, in both care settings, care time did not include informal care. As both attendance in day activities and informal care can reduce formal care needs and enable living at home, the present definition of care time must be considered when interpreting the results. Lastly, due to the cross-sectional study design, causality cannot be inferred from the results.

## Conclusions

Functioning in activities of daily living was the strongest predictor of received care time in both care settings. Therefore, it is essential to maintain or even improve physical functioning to restrain the increasing need for care resources. Care needs of older people in home care and assisted living facilities are often complex, as factors such as cognitive decline, depressive mood, and pain often appear simultaneously, affecting physical functioning. Therefore, in addition to rehabilitation, there may be other potential interventions that improve functioning, including social activities. The use of autonomous teams should be explored further to aid in productivity and quality of care. Along with restraining resource use, it is crucial to ensure that every client receives care according to their holistic care needs.

## Data Availability

The datasets generated and analysed during the current study are not publicly available due the fact that they constitute an excerpt of research in progress but are available from the corresponding author on reasonable request.

## Abbreviations

ADL-H: Activities of Daily Living Hierarchy -scale
CHESS: Changes in Health, End-stage disease, and Symptoms and Signs -scale
CI: Confidence Interval
COVID-19: Coronavirus disease
CPS: Cognitive Performance Scale
DRS: Depression Rating Scale
ERP: Enterprise Planning System
GDP: Gross domestic product
IADL: Instrumental Activities of Daily Living Capacity -scale
ICC: Intraclass correlation
MAPLe: Method for Assigning Priority Levels
PAIN: Pain indicator
RAI: Resident Assessment Instrument
RAI-HC: Resident Assessment Instrument Home Care tool
SD: Standard Deviation
WHO: The World Health Organization
